# Urban Indian healthcare referral system: A qualitative exploration from the physicians’ perspectives

**DOI:** 10.1371/journal.pone.0338113

**Published:** 2025-12-04

**Authors:** Maria Sabastin Sagayam, Priya Gupta, Ram Ramesh, Angan Sengupta

**Affiliations:** 1 Amrita School of Business, Amrita Vishwa Vidyapeetham, Bengaluru, India; 2 University at Buffalo, State University of New York, Albany, New York, United States of America; University of Kwazulu-Natal, SOUTH AFRICA

## Abstract

**Background:**

The Indian healthcare system continues to remain unstructured leading to sub-optimal health outcomes, not just in rural but even in urban areas. While physicians play a crucial role in shaping treatment trajectories and managing the referral process, their perspective on the referral system has received very limited academic attention in India. This study attempts to understand the archetypical physician’s referral mechanism and the factors influencing their referral practices. This study also highlights the challenges and possible solutions in operationalising an efficient referral process as suggested by the professionals.

**Methods:**

In-depth qualitative interviews were conducted with sixty-two physicians consisting of both general physicians and specialists from 19 different disciplines, associated with public and private hospitals in Bengaluru, India. The data, thus collected, was subjected to thematic analysis to generate relevant themes.

**Results:**

Five themes emerged from the thematic analysis from a phenomenological perspective based on the physicians’ lived experience. First of all, specialist physicians’ availability, accessibility, experience, and reputation strongly influenced referral recommendations. It was also observed that due to lack of a comprehensive healthcare provider database, personal connections and professional networks are utilised. Moreover, although physicians prioritize patients’ affordability and accessibility factors, referral counselling and caregiver-patient communication remained inadequate and required formalization. While the fourth theme clusters around several barriers related to communication, system inefficiencies, lack of awareness, accessibility and affordability among patients; the final theme suggests that the physicians emphasized on urgent need for clear guidelines, regulations and policies to streamline and monitor the referral system.

**Conclusion:**

This research highlights that physicians recognize the systemic gaps leading to unsolicited health outcomes; yet they are helpless in most cases. The participants emphasized that robust information systems connecting all relevant stakeholders are essential. The exploration reveals that the system will not adopt a structured referral method without the government taking interest in it.

## Introduction

India, one of the most populous countries in the world, faces a range of complex and diverse healthcare challenges [1]. India’s health system, underwent significant changes while aiming to achieve Universal Health Coverage [2]. Yet, challenges persist in ensuring accessible and efficient healthcare for all citizens at affordable cost [3]. A major concern is the severe shortage of trained medical practitioners [4,5] aggravated by a fragmented healthcare system and inadequate regulatory frameworks [6]. This leads to systemic gaps that hinder effective patient-provider connections and ultimately contribute to suboptimal health outcomes.

The ineffective referral process can be identified as one such gap which plays a central role in ensuring continuity of care. The World Health Organization (WHO) defines referral as the act of seeking support from a better-resourced facility, when a health worker lacks the capacity to manage a patient’s condition [7]. The referral process, an essential component of a well-functioning healthcare system, is particularly critical in bridging the gaps between patients, general physicians and specialists [8], ensuring timely and cost-effective treatments [7]. A referral system, equipped with a strong database helps healthcare providers identify and collaborate easily with specialist caregivers [9]. A narrative literature review has highlighted that patients often face significant delays in accessing specialized care due to inefficient referral pathways and lack of streamlined processes [10]. Another scoping review noted that challenges such as lack of follow-up care, inadequate patient education about referral processes, and the financial burden associated with referrals have further compounded the issues for patients [11]. However, in India, the referral process remains poorly structured and inadequately regulated, leading to significant inefficiencies in patient care [7,12,13]. For instance, a study highlighted that inadequate referral mechanisms often aggravate delays in treatment and increase the burden on tertiary care centers [9]. Similarly, another study mentions that the lack of streamlined referral pathways in obstetric emergency care contributes to poor health outcomes and patient dissatisfaction [12]. However, physicians play a pivotal role in the referral process, serving as gatekeepers to specialized care thereby, ensuring that patients receive timely and appropriate interventions [14]. They also maintain continuity of care by providing detailed communication to specialists, bridging the gap between primary and tertiary care, and avoiding disruptions that could lead to fragmented treatment [15]. At the same time, physicians’ inefficiencies lead to unnecessary diagnostic suggestions and delay in referrals [16].

Studies from other countries have tried to understand the factors associated with the gaps in the referral process from physicians’ perspectives. A study based on physicians in Boston observed that even if primary physicians adhere to the referral system, the specialists do not [17]. Physicians and experts in Austria stated that financial constraints can make it hard for people to access healthcare, especially in the case of expensive consultations [18].

In India, rural and urban healthcare scenarios differ considerably, as do their referral systems. A few studies have tried to examine the limitations in referral mechanisms in rural and remote areas. However, there is a paucity of research on the informal nature of the referral system in urban India, particularly from physicians’ perspectives. Several factors lead to improper referral decisions by patients, resulting in inferior health outcomes in urban India. Therefore, this study aims to address this critical gap by exploring the factors influencing referral decisions, communication methods, treatment management, patient adherence, and health outcomes from physicians’ viewpoints. The need for this study is underscored by the growing recognition that an effective referral system can significantly improve healthcare delivery and outcomes, even in urban areas. Specifically, understanding the challenges faced by physicians while referring patients can provide actionable insights to enhance the referral process. This study aims to influence policy and practice by providing a foundation for targeted interventions and evidence-based policy measures.

### Literature review

This section offers a synthesis of existing literature highlighting on limitations of healthcare referral system in India, primarily focusing on the urban studies, irrespective of their targeted respondents whether they are physicians or patients. In India, the healthcare system is characterized by a hierarchical referral structure, wherein primary care physicians serve as gatekeepers, facilitating referrals to specialists as needed, regardless of rural or urban settings [13]. However, in practice, adherence to healthcare access and referral procedures has been questionable and shows significant variation between rural and urban landscapes in India [19]. In rural areas, formal healthcare is primarily delivered through public facilities. The health seeking behaviour of rural residents are generally constrained by the limited availability of healthcare providers. They are compelled to adhere to the referral suggestions [20]. However, the scenario is different in Indian cities.

The public health infrastructure in urban area is designed by following similar hierarchy. Even though National Urban Health Mission (NUHM) was launched in India, it failed to strengthen the primary health centres and state governed referral system [21]. Urban areas are insufficiently served by public funded hospitals [22], and they are often crowded by urban poor [21]. Adherence to referral procedures has neither been a requirement nor a mandate in urban landscapes in India. In urban areas, private sector plays key role in offering healthcare services at all levels, and a large number of independent caregivers operate within an unstandardized system characterized by inconsistent referral criteria. Urban patients are increasingly preferring private healthcare providers [23]. While there is a tendency to bypass primary healthcare centers (PHCs), urban poor are the ones who are benefitted by the public facilities [24] and the rich visit the private healthcare providers. The tendency to bypass primary and secondary care facilities is further compounded by the easy availability of private specialists in urban India [12]. Many patients in urban India do not utilize public healthcare facilities due to limited awareness of their availability and a lack of trust in the quality of care provided [20]. The Outpatient Departments (OPDs) in public-funded tertiary care facilities are often overcrowded with patients seeking treatment for minor ailments that could be effectively managed at the primary healthcare level [9]. Yet, even in urban India, there is a suboptimal ratio between specialist physicians and the population [25].

Physicians often cite multiple factors when referring a patient to another caregiver. These include personal relationships with other professionals and their networks, as well as patient’s affordability to avail services from certain caregivers [17]. Limited communication and information exchange between referring and receiving facilities impede seamless patient transfers and continuity of care [12]. Due to the unstructured referral system, there is information asymmetry and information overload among the patients and physicians regarding the availability of specialist physicians, often leading to inefficient decision-making. Patient confusion as well as inconsistent practices among healthcare providers, contributes to patients’ intentions to switch providers and to avoid referrals by physicians [26,27]. Patients’ adherence to referral recommendation, and continuity of care often depend on the quality of the physician-patient relationship and patients’ satisfaction [26,28]. While studies have advocated for advancement in health information management systems [29], addressing these barriers requires comprehensive reforms, including policy changes, investment in infrastructure, capacity building, and community engagement, to enhance the efficiency and effectiveness of the referral process, ultimately improving health outcomes and reducing disparities across the population.

While designing policies for an efficient referral process, healthcare professionals and policymakers must understand the system, knowledge, perceptions, and practices among various stakeholders [21]. Given this background, this study explores referral decision-making practices by physicians in an urban healthcare system in India and identifies the factors that influence the current healthcare referral system.

The two major research questions that are addressed in this study are:

1. What are the archetypal physician referral mechanisms and the factors influencing referral practices in the urban Indian healthcare system, from the physicians’ perspectives?
2. What are the challenges and the scopes of improvement in the existing urban Indian healthcare system as perceived by the physicians?

Addressing the above stated questions, this study aims to explore clinical, social, economic, psychological, and organizational factors that influence referral decisions. Given a dearth of studies delving into the referral mechanism as a key barrier to efficient healthcare delivery in India, the authors hope to add to the corpus of knowledge on physician referrals in the urban Indian healthcare system that will benefit the policymakers, healthcare executives, and researchers in formulating a standardized and efficient healthcare referral system.

### Theoretical underpinning

This study employs the Health Belief Model (HBM) as a guiding theoretical framework to elucidate our findings. The HBM, was originally developed to predict and explain how individuals decide to take specific health-related actions, such as seeking medical care or adhering to treatment based on individuals’ assessment of their health risks and the potential outcomes of their actions [30]. The decisions are taken based on four key factors: (i) perceived severity, (ii) perceived susceptibility, (iii) perceived benefits, and (iv) perceived barriers. Perceived severity refers to the seriousness of a condition, while perceived susceptibility is the likelihood of experiencing the condition. Perceived benefits and barriers are the positive outcomes of acting and the obstacles that might prevent from acting, respectively. However, the core principles of this model can also be extended to analyse how physicians make referral decisions, based on these attributes. Methodologically, this study adopted a Phenomenological approach to understand and interpret how physicians make referral decisions.

### Methodology

This study used a qualitative research approach, primarily a phenomenological approach, to understand the physicians’ referral practices and the factors impacting referral choices within the urban Indian healthcare system. Phenomenological research helps us understand participants lived experiences and capture the inherent meaning and essence each experience carries, which is captured through thematic analysis [31]. Through this method, we expect to uncover and understand physicians’ experiences, perspectives, and decision-making processes within the context of referral systems.

#### Participants.

A total of 62 doctors from various specializations, healthcare settings, and demographic backgrounds participated in this study. The interviews were conducted in urban Bengaluru, the capital city of Karnataka state. Bengaluru, with a population of 14 million, is widely recognized as the Silicon Valley of India. The state is well known for its advanced medical facilities and is a highly sought-after healthcare tourism destination, catering to many patients. Out of 309 private hospitals, 25 diagnostic centers, and nine ayurvedic and naturopathy hospitals have been recognised by the state government for medical reimbursements [32].

Table 1 presents the sample distribution based on certain important background characteristics. Two-thirds are females, while three out of four were exclusively attached to private healthcare institutions. Twenty-seven percent of the participants have more than twenty years of experience.

**Table 1:** The sample distribution.

Category		No. of Physician	%
Gender	Male	40	64.5
	Female	22	35.5
Type of Healthcare institution	Private	49	79.0
	Public	6	9.7
	Both	7	11.3
Clinical Experience	Less than 10 years	7	11.3
	10 to 20 years	33	53.2
	21 to 30 years	16	25.8
	More than 30 years	6	9.7
Specialization	General practitioner	10	16.1
	Medical specialist	52	83.9

#### Data collection.

An ethical clearance was procured from the Amrita Vishwa Vidyapeetham’s Institutional Review Board (IEC-AIMS-2021-DOM-172). The first author collected the data between December 2022 and April 2023. Purposive sampling and snow-balling sampling techniques were followed to select participant doctors who have been involved in referral process. Prior appointment was taken before each interview by either directly calling or texting the doctors, or by contacting their secretaries. Consent was obtained from all the physicians before each interview. They were asked to read and sign the consent form upon approval. The consent form stated the objectives of this study, alongside confirming the anonymity, confidentiality and non-commercial intent of the data or the results. To accurately capture the nuance of the participants’ comments, the interviews were audio-recorded only with their permission. The transcripts and audio recording were stored in a password protected drive and in the personal cloud. The access to the original data was only retained with two of the authors.

In-depth interviews were conducted using an open-ended data collection tool. The duration of the interviews ranged between 35–70 minutes. The attributes of data collection tool were drawn based upon the constructs studied under Health Belief Model. The questions attempted to explore the physicians’ perceived susceptibility of a certain case that requires further referral; perceived severity of the case which may aggravate to a serious condition if the referral process fails; perceived barriers that the stakeholders face towards suggesting a referral and completing the process while referring a patient to a specialist physician or any other facility, and communicating the perceived benefits derived from the referral. This study also tried to explore the cues that the physicians endorse to ensure referral adherence and improving self-efficacy among patients. The questions were designed carefully to ensure participants felt at ease without any inhibitions, while sharing their opinions, life experiences or their understanding of the referral systems as well as the factors influencing the referral decisions. Interview schedule consisted of questions pertaining to when and how physicians initiate referrals, patients’ reactions to referrals, post-referral scenarios, and the personal or professional influences impacting referral decisions. Additionally, it addressed obstacles within the current referral process, the supportive networks aiding in referrals, collaborative efforts among physicians in facilitating referrals, patient-physician relationship, potential enhancements to the existing system, and technological advancements enhancing referral efficiency. Verbatim transcription of the audio files were undertaken to generate textual data. During the process of data gathering and analysis particular attention was paid to data saturation. Although saturation of receiving new information was reached by sixtieth interview, to ensure no new information may be missed out, two more interviews were conducted, bringing the final count of respondents to sixty- two.

#### Data analysis.

The interview data were analyzed using thematic analysis to systematically recognize the data’s recurring patterns and thereby identifying the themes and categories. Initially, researchers familiarized themselves with the data by thoroughly reviewing the transcriptions. Subsequently, a coding was conducted, identifying meaningful data units and assigning relevant codes. MS excel was used to store and code data manually by researchers. These codes were then collated and organized into broader themes that captured the essence of the data. Throughout the analysis, codes and themes were reviewed, refined, and validated through discussions among the researchers to ensure accuracy and consistency.

### Identification of key themes

**Fig 1:**
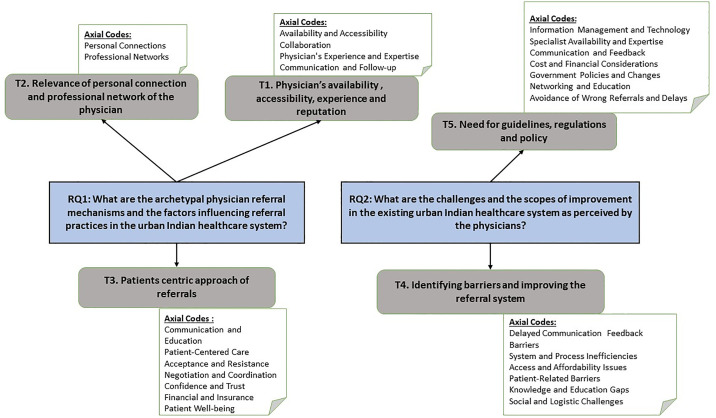
Axial codes and development of themes.

**Fig 2:**
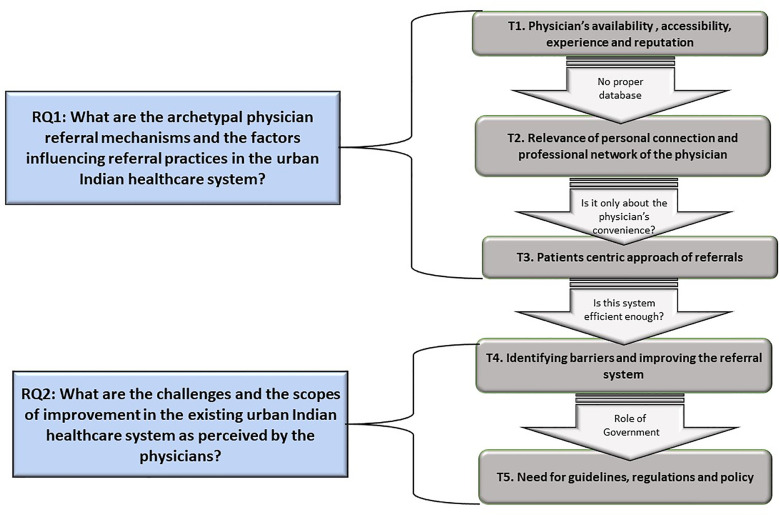
Thematic analysis framework.

The analysis has resulted in five key themes. The framework depicting the theme generation using the axial codes are stated in Fig 1. The Fig 2 presents the logical arguments connecting the themes.

#### Theme 1: Physician’s availability, accessibility, experience and reputation.

Even in the absence of a well-established referral mechanism physicians do not make arbitrary choices when deciding on referrals for their patients. The availability of a doctor is often the most crucial factor. However, without a structured referral system in place, they face significant constraints in identifying the available physicians.

“*The current referral process from primary care to specialty care has several weaknesses like identifying the right specialist, duplication of tests, multiple medications taken for the same health condition, which leads to deterioration of patient health”* – *Reported by Physician from private hospital*

In emergency cases, when referrals must be made to another care provider, without any institutional mandate on infrastructural support, often accessibility appears as a serious challenge. When specialists are not available in the healthcare facility visited by the patient, physicians typically ask patients about their location and suggests referrals based on patients’ geographical convenience.

“*If ambulance services are not immediately available it is very difficult for patients to travel little far and avail the healthcare in critical cases”- Mentioned by an orthopaedic doctor*

Additionally, experience is valued highly among physicians. The experience of physicians not only enhances a physician’s diagnostic and treatment skills, but also helps develops the professional network, which increases the knowledge about availability and accessibility of physicians to be referred. Experience also instils confidence in both the referring doctors and the patients being referred, as well as patients to be referred.

*“I generally consider the experience of the physician. Because experienced specialists diagnose and treat patients better”*- *Reported by Physician from private hospital*

When making referral decisions, the reputation of the medical specialists to whom patients are referred is also crucial. Personal experiences, whether encountered personally or relayed through colleagues, hold immense weight. Physicians rely on these experiences to gauge the quality of care offered by referral hospitals. In absence of an exhaustive database and regulated referral framework, physicians generally trust peer reviews and take help of their networks to create individual level database of availability of experienced and reputed physicians across geographies.

*“The physicians who are more effective, ethical and skilled are preferred.”- As told by a physician from multi-specialty hospital*

*“Generally, only low-income people come to me for treatment; so…before referral, I try to understand whether the physician is accessible and affordable. Also he/she (sic) is knowledgeable and well known in the public.”- As mentioned by a general physician*

#### Theme 2: Relevance of personal connection and professional network.

The open codes derived from the data highlighted the relevance of personal connections and professional networks in the physicians’ decision-making process. These codes encompass a wide range of avenues through which physicians establish and nurture these relationships, influencing their referral decisions.

Ethical practices and the skills of medical professionals are key factors that contribute to patients’ trust in healthcare services. In India, referring doctors often feel that when they refer their patients to physicians within their professional and personal networks, they can convince their patients by effectively justifying the referral, even if it evades the formal protocol.

*“Patients rely on us to guide them to the right specialists. It’s not just about medical expertise; it’s about ensuring the patient feels comfortable and confident in the care they’re receiving. Personal relationships play a significant role in that.”- As mentioned by a physician with 35 years of experience at different facilities*

Maintaining open lines of communication with colleagues at referral hospitals paves the way for more effective care transitions and continuity of care through close coordination, especially when they share an affable relationship. Maintaining companionship with current and former healthcare colleagues is essential. Modern communication platforms such as WhatsApp and other social networks play a crucial role in nurturing peer communication. In certain cases, personal recommendations from professional acquaintances, local associations and area-specific contacts play a notable role in taking a referral decision and shaping the referral process.

*“Peer recommendations have been a game-changer for me. When I am uncertain about a referral, I reach out to specialists that my colleagues have recommended. It’s like having a reliable resource right at my fingertips.”- As mentioned by an oncologist at a private facility*

In addition, an atmosphere that promotes the sharing and discussion of cases, contributes to the development of collective learning and expertise, while an environment that fosters collaboration based on the nature of the disease enables the development of treatment strategies that are comprehensive and well-coordinated. In India referral networks are also developed through interactions with hospital and pharmaceutical company representatives and specialized associations. In addition, local and regional associations, specialty groups, and medical council meetings provide platforms for building such connections. Physicians participate in medical conferences to expand their networks, foster professional growth, and stay updated on new research and technology.

*“Being a part of medical conferences has been incredibly valuable for me. The relationships I’ve built with experts from various fields have made referrals smoother. There’s a level of trust and understanding that comes from these interactions, and it benefits the patients”- As mentioned by a senior physician from private healthcare*

#### Theme 3: Patient-centric approach of referrals.

Our participants opined that for a referral to be successful, physicians should provide comprehensive explanations regarding the ailment, reasons for referral, potential benefits, and any associated risks or alternatives. Patients must be convinced about the referral being suggested. Educating them about their conditions, treatment options, and expected outcomes empowers them to make informed decisions.

*“Patient satisfaction depends on how well we explain why referrals are needed and deal with things like insurance. It’s important to talk clearly about these things to make the process smooth.”- Mentioned by a cardiologist*

Patients’ level of awareness influences the referral trajectory. Treatment success can be improved through disease-specific knowledge combined with routine follow-ups. Patient reviews and experiences serve as valuable touchpoints in shaping the referral process. Physicians draw insights from patients’ feedback and experiences, and often make recommendations based on patients’ satisfaction level, feedback on clinical proficiency, quality of care, and communication of the referred doctor or medical facility.

*“As a doctor, I find patient feedback and experiences to be very helpful. When I’m thinking about who to suggest, good reviews can help me decide. Knowing that other people have had good experiences is comforting, and it gives me faith in the reference process.”- As mentioned by a Senior surgeon*

When making treatment recommendations, referring doctors consider patient’s willingness and ability to pay for the recommended doctors’ consultations, hospitalisation, diagnostics, and other treatment related costs. Sometimes basic interactions with patients provide referring doctors cues about their economic status and awareness level, based on which they decide whether to treat the patients themselves or to refer them to another facility.

*“I generally consider my patients’ opinions before referral trying to understand how well they are financially supported, whether they will be able to afford the treatment at the referred hospital or not? Not only me, but most of my colleagues do also consider these circumstances before referral”- As told by a general physician from private nursing home*

“*Ours is a small facility, unable to take care of immediate actions and complicated cases, especially at odd hours when we are short of staffs. I do not take risk when patients’ family members are unwilling to wait, lack knowledge, and unable to assess the situation. If I realise that they are coming from poorer economic background, I refer them to the charitable low-cost tertiary care hospital (6km away from the facility)*”- *As stated by an emergency doctor from a small nursing home*.

Insurance coverage status of patients is also enquired by the physicians while suggesting further treatments. Insurance reduces out-of-pocket costs and increases medical access. The physicians additionally check whether the patients are beneficiaries of any government sponsored health related schemes and try to identify referral institutions accordingly.

*“Insurance plays a major role when it comes to referral and unfortunately most of the low-income people will not have proper health insurance. I thoroughly understand my patient’s condition and their income level before referral and at the same time if any government scheme like Ayushman Bharat- Arogya Karnataka Scheme can be helpful, and I recommend my patients accordingly”- As mentioned by physician from public healthcare*

Insurance complexities can deter patients from pursuing referrals, necessitating careful navigation of the system. Treating physicians cannot always validate the scope of the insurance schemes and its applicability for individual cases and care provider. Patients obtaining treatment from approved providers may have to pay co-payments, deductibles, or fees for procedures not covered by insurance or government-sponsored health schemes.

*“Monetary issues. Patients don’t want to spend on health. They want best treatment with least expenses. Saving money is more important than getting the best possible treatment.”- Mentioned by physician from public healthcare*

Adherence to a referral suggestion is better for patients who easily accept referrals. Conversely, challenges arise in cases where patients resist referrals due to several. The referring doctor is required to assess the factors that can influence an individual patient’s satisfaction with a referral suggestion, which might impact their referral compliance.

*“Moreover, referral satisfaction is essential for routine follow-up and better treatment.”- Mentioned by a gynaecologist*

*“However, we must address resistance to referrals stemming from trust, financial concerns, or personal anxieties.”- Stated by a general physician from private healthcare*

At the core of the referral process lies patient’s well-being, which should be the primary focus throughout the journey. The referral process should prioritize the patient’s needs, preferences, and values. A patient-centered approach ensures that care aligns with individual goals and expectations, and regular follow-up appointments and monitoring of patient progress post-referral are essential. This ensures that patients receive the necessary care and support throughout their healthcare journey.

*“The priority is the patient’s well-being. A patient-centered approach is non-negotiable. We must prioritize their needs, preferences, and values to provide the best possible care and support throughout their healthcare journey.”- As mentioned by a physician from private healthcare*

#### Theme 4: Identifying barriers and improving the referral system.

The identification and resolution of obstacles within the referral system, as perceived by referring doctors, are essential for enhancing the provision of healthcare services. The following section discusses the barriers faced while making referrals and the improvement possibilities as identified by referring doctors, based on important axial codes generated through our analysis.

##### Sub -Theme 4.1 Identifying Barriers:

The respondents rued that there is no uniform communication standard in the Indian healthcare industry. Delays in the delivery of medical treatment are often caused by systemic and procedural inefficiencies. Many referring physicians struggle to find available specialists, particularly in underserved areas and critical cases. There have been instances when specialists do not respond or reply very slowly, or do not send complete information of patients’ status. Referring physicians themselves face unique challenges, including gaps in specialized knowledge and concerns about losing patients to specialists. At the same time, financial barriers for patients impedes and deters appropriate referrals.

*“Time is a barrier here. Often once we refer the patient to some other hospital, the time taken by the concerned consultant to see the patient is very long”- Mentioned by physician from private healthcare*

In Bengaluru, public health facilities cover many specialized requirements. Yet in the absence of a strong primary healthcare system otherwise, physicians from tertiary care public health facilities complained about crowding of too many patients without any referral history. Nevertheless, irrespective of the type of facility, physicians serving in tertiary care hospitals are over-stretched.

*“Too many patients arrive here without any proper information from the general physicians”- As told by a physician from a tertiary care government hospital*

Patient-related barriers, such as non-adherence and limited health literacy, further complicate the referral process. Smaller facilities, which are not equipped enough to treat complicated emergency cases, tend to refer patients to larger facilities even while facing much trivial cases, fearing mob violence. Such apprehensions are mentioned by the physicians at public tertiary care hospitals as well, where doctors treat mainly underprivileged sections of the population. Due to unawareness of the patients and their families any delay in the referral or treatment process may lead to serious consequences.

*“Knowledge and education gaps among referring physicians, coupled with social and logistic challenges like transportation issues and language barriers, can be addressed through ongoing training, technological support, and through telemedicine options.”*- *As said by a general physician*

#### Theme 4.2 Improving the Referral System.

Referring doctors, referred specialists, and administrative staff of a health facility are usually involved in the referral process, alongside the patients at the receiving end. Streamlining this complex process requires effective negotiation and cooperation among the stakeholders that guarantee timely referral, efficient exchange of patient data, smooth handoffs, and finally a coordinated treatment.

*“Effective communication between referring physicians and specialists is the backbone of our referral system. Timely feedback ensures that we can provide our patients with coordinated and efficient care.”- As reported by a senior cardiologist*

Referring physicians navigate a multifaceted landscape of challenges within the healthcare referral system, with each challenge presenting unique opportunities for improvement, especially with the advent of modern technologies. An integrated database management system will enhance knowledge about the availability and accessibility of specialists. Advancement in information management and technology is mandatory to ensure seamless exchange of crucial patient data, in turn reducing the turn-around-time significantly.

*“Managing patient information can be a real struggle in our referral system. Outdated technology and interoperability issues often lead to delays and inefficiencies in the exchange of critical patient data.”- Mentioned by an allergy specialist*

Nine participants out of 62 (14.5%) emphasized on security of patient data should not be breached in this process. The ethical integrity of the stakeholders, and the robustness of the system must be closely monitored.

*“Patients’ data needs to be shared in a comprehensive referral system. At the same time, it must be safe and secured.”- As mentioned by a physician from super-specialty hospital*

The presence of a variety of medical specializations within the same institute or an extended professional network, broadens the spectrum of medical services, thereby meeting the requirements of treating complicated cases and a larger number of patients. Expanding specialist access in underserved regions and addressing affordability through insurance reforms or financial assistance programs can help alleviate these concerns. Twenty-one percent of the study participants stated that continuous medical education and collaborative care models can bolster referring physicians’ skills and confidence. At the same time, seven physicians opined that counselling the patients during referral, increasing patient awareness of the treatment trajectory and better communication strategies involving physicians at both ends, support staff and patients can enhance patient adherence to referrals and referral outcomes.

*“Our schedules are packed from morning to evening; following up on each referral just isn’t practical under these conditions “ - As said by a physician from super specialty hospital.*

Seven out of 52 specialists (13.4%), out of which two were working in public, four in private hospitals and one in both types of facilities, complained about high workload and claimed this to be a prime reason for not being able to monitor whether the suggested referrals were being followed. Crucially, avoidance of incorrect referrals and undue delays are top priorities, as referring physicians strive to ensure that the initial referral is precise. Participants felt that if the diagnosis or treatment process starts from family physicians and if the primary care physicians are empowered and educated to manage a treatment trajectory, referrals can be more effective. Additionally, it is essential to recruit dedicated staff for management of referral cases at healthcare institutions.

#### Theme 5: Need for guidelines, regulations and policies.

As lack of modern tools and limited utilization of technology have emerged as important barriers for effective referral (refer to section 4.2), the participants, while suggesting policy recommendations strongly advocated towards mandating of use of electronic health records and efficient scheduling algorithms especially for public healthcare facilities.

The physicians opined that the policymakers must design and mandate a standardized and structured referral mechanism for all healthcare institutions and individual physicians. Establishing and enforcing uniform communication standards is crucial in this regard. The system should be regulated and monitored at regular intervals by the authority. Quality control department, involving hospital representatives, should include referral practices under its agenda. Since the information and support provided by non-clinical staffs to both physicians and patients in healthcare institutions significantly influence the referral process, clear guidelines should be framed for such human resources ensuring that they communicate relevant details effectively, aiding physicians in making informed decisions. More focused training programs on referral systems and regulations are needed to alleviate the strain on higher-level healthcare facilities strengthening the referral mechanisms.

*“Clear rules and standards make it easy for hospital staff to share important information. Support services help me set up appointments, and do other routine jobs, which gives me more time to care for patients.”- As mentioned by a pediatrician*

By streamlining the referral process and easing administrative duties, support services from the non-clinical personnel allow referring doctors to concentrate on giving their patients high-quality care. In an integrated framework, while referring physicians would technically collaborate with referred healthcare professionals, the administrative staffs should establish an easy referral process and facilitate transfer between referring and referred healthcare institutions by adhering to the guidelines and policies.

## Discussion

This study, while studying practicing physicians’ experiences in Bengaluru, summarizes the difficulties faced by our participants, or physicians in several other cities might be facing, due to the unstructured and under-regulated network while revealing areas for improvement. This study could identify several crucial factors that affect the efficiency of the referral process, such as the timely availability of physicians or specialists, accessibility, cost considerations, and poor professional networks of healthcare professionals. Although referral decisions are largely patient-centric, compliance with referral suggestions can be improved through proper communication between caregivers and patients, patient counselling, collaborative decision-making, and integration of patient input.

While Health Belief Model is mostly studied from patients’ perspective, this current study has adopted a novel approach by assessing physicians’ perspective through the lens of HBM. Theoretically, in the context of the findings of this study, HBM is found to be relevant; because it offers a structured way to explore the cognitive processes that physicians undertake when deciding whether to refer a patient for specialized care and where. The physician should assess the patient, clearly communicate the risk level for a certain condition, and why further referral is required (perceived susceptibility). The physicians should also inform the patients about the potential consequences of the condition if the referral suggestions are not followed (perceived severity). They also need to explain the potential advantages of the referral to a specialist, such as improved diagnosis or treatment (perceived benefits), and about possible obstacles like limited specialist availability, cost, anxiety, logistics challenges etc. (perceived barriers), which collectively guide the final decision. Cues to action can be indulged by insisting on the urgency, introducing written formal referrals, personally connecting the patients to the referred physicians and by maintaining a follow-up. Finally, increasing patients’ confidence in adhering to the suggested referral though proper communication should address the construct of ‘self-efficacy’. It is essential to approach the problem theoretically because it allows for a standardised adoption of programs and policies and gives room for nuanced analysis of how and why certain referrals are made, ultimately identifying areas where interventions may be needed to improve the referral process.

Similar to our study finding, earlier studies also suggested that there needs to be efforts to enhance the efficiency of the healthcare referral system in India that include strengthening of primary care [9], standardizing referral processes, and improving communication and coordination between primary care physicians and specialists [15]. Accessibility of specialists present significant challenges [17]. As has been observed in our study, earlier studies as well have identified that personal relationships between primary care physicians and specialists and their engagement in professional networks influence referral patterns, especially in situations requiring specialized competence [17]. Our study findings corroborate synthesis of a systematic literature that examined research manuscripts published between 2000 and 2021, while it observed that in USA physicians use their professional networks, personal ties, and patients’ feedbacks based on their interactions with the referred doctors, to assess the clinical expertise and reputations of the specialists before making referrals [33].

The current study suggests referring physicians should share medical records and test results with the specialist. Gandhi et al. (2008) suggested that to ensure a smooth transition of patients and continuity of care, clear and concise patient data must be shared between the referring and the referred physicians, and collaboration must be encouraged between healthcare facilities for a cohesive patient care approach [34]. Even in USA, referring doctors often encounter communication issues from the referred specialists when opening a referral process, or following up on patients’ progress [35]. As suggested by O’Daniel and Rosenstein (2008), the experiential account of the caregiver participants also indicate that effective communication and teamwork among healthcare professionals leads to continued improvement in decision making and assists in carrying out plans for patient care [36].

Lack of referral knowledge does not only impact the healthcare outcomes in resource constraint systems, but the same has also been observed in developed countries. A study conducted in pregnancy and maternal care in USA highlighted that physicians who were willing to consult specialized obstetricians and gynaecologists, were unable to refer patients because of their lack of knowledge about the availability of the specialists arising from inadequate training [37]. Our observations reinforce prevalence of such knowledge barriers in India as well. A recent study conducted in urban India has observed that family physicians play a major role in determining health-seeking behaviour of patients that include pattern of consultations, hospitalization and referrals [38]; and hence might potentially remove the knowledge gaps at the onset of the treatment trajectory. Since, family physicians initiate referral for further treatments, it has been suggested that family physicians be trained and equipped with knowledge on the specialists’ networks both in rural and urban India [39]. Even though our study, while recommending provider training and robust documentation, has not specifically focused on any specialisation, a study conducted in India highlights sub-optimal knowledge among providers regarding high-risk pregnancies and inadequate documentation and communication result in unnecessary referrals and gaps in the referral process for patients with obstetric emergency particularly from private facilities [12]. Our study suggests that unless the system ensures ease of information sharing among the stakeholders, only formalizing providers’ networks will remain insufficient. This argument finds support from an earlier systematic review which endorses effective teamwork and streamlining access to patient information among healthcare providers, both within and beyond institutions, which is crucial for enhancing patients’ confidence, improved care coordination and quality, informed referral decisions, and reduction in unnecessary procedures, and improve patient confidentiality [40].

This study has emphasized that even though the referral decisions are patient-centered, there is a need of counselling and better communication between the caregivers and the patients, especially when patients are unaware of their ailment, they are not satisfied and economically underprivileged. The patient-physician relationship is now widely recognized as a valuable strategic marketing tool for healthcare providers and services [28]. Authors found that the quality of the patient-physician relationship, specifically one that is distinguished by collaborative decision-making and open communication, can have a positive impact on the degree to which patients comply with treatment and referral recommendations [41]. Earlier studies identified that in India as well, physician-patient interaction plays a critical role in patient satisfaction, retention and referral compliance [26,42]. Echoing our results, another study focusing on cancer care in India strongly corroborated that the involvement of patients and their family members in decision-making and the consideration of their needs, concerns and socio-economic factors are crucial aspects of patient-centered care [42,43].

This is an established fact that high out-of-pocket costs significantly hinder healthcare access in India [44]. The participants of this study have urged on improving healthcare affordability for poor people through state sponsored insurance coverage, since they observe that patients’ compliance to referral suggestions and continuation of treatment depends on the financial burden associated with the referred treatment. Similar to the observations from a previous study, physicians participating in the current study has also faced challenge of reconciling the need for specialist medical treatment given the current and expected financial burden [17]. Furthermore, a suggestion that emerges from this study is, while referring patients to another healthcare provider, sharing information on their insurance status and coverage can help mitigate financial dilemmas, reduce procedural delays and improve access to care.

Our findings highlight that a formal communication and feedback system between referring and referred caregivers is an utmost necessity. This aligns with evidence from a study on stroke patients in India that suggests a written Standard Operating procedure (SOP) on inter-hospital transfers will facilitate the timely care [45]. Another multi-site study across rural, peri-urban and urban public hospitals in India observed that irrespective of the location of the hospitals, discharge summaries were often unstructured, in many cases verbally delivered, or hastily scribbled onto cramped prescription forms leading to suboptimal health outcomes, loss of follow-ups and, in some cases, death [46]. The state government sponsored Mohalla Clinics in Delhi, which specifically serves slum dwellers, were not found to be systematically connected even with public funded secondary and tertiary care institutions for referral purposes [24].

Effective referral system management requires government investment and policy planning, including infrastructure improvements, enhanced communication and transportation, and standardized monitoring. However, opaque government policies and regulations create challenges related to advocacy by the state functionaries, adherence to the norms by the healthcare institutions, technology adoption, and data share [11]. As it was observed in Lebanon, new models for collaboration, such as streamlined referral processes and shared information systems, could help in patient identification, enhance patient care and foster a more equitable system in India as well [47]. Similarly, identifying barriers and implementing technology-based interventions like teleconsultations, and improved communication platforms can provide quality and affordable healthcare to poor people of India [29]. Technology can bring noteworthy healthcare solutions for slum and non-slum people, though distinctly, in urban India [38]. Moreover, findings from a study conducted in a developed country (Netherlands) noted that even though electronic health records can facilitate joint clinical decision-making based on shared patient data, it can also be constrained by increased administrative load for the physicians [48]. Hence, a country like India which already suffers from a fragmented system and poor physician-patient ratio, the technology adoption in the referral mechanism must be methodically executed.

This study offers a very important observation that none of the physicians providing health care in a metro-city of India, stated infrastructure as a reason for inefficient referrals. However, a recent study conducted on emergency obstetric care in public health facilities of urban Maharashtra reported that besides the noteworthy absence of sufficient anaesthetists, paediatricians, obstetricians, and other physicians, need for betterment of infrastructure, such as emergency, Operation Theatre, and Neonatal Intensive Care Unit, can be cited as another critical reason for emergency referrals [49].

Ultimately, the healthcare systems that are serving people in remote areas, rural population, urban rich and urban poor, are not only distinct, but considerably disintegrated in absence of referral linkages. Lack of systemic interconnectedness causes delays in accessing specialized care for those who have limited accessibility and affordability; and these socio-economic and regional disparities can be addressed effectively through a strong healthcare referral mechanism [49,50]. A recent study proposes a streamlined approach to improve the rural healthcare system by reimagining the flagship telemedicine program of government of India named eSanjeevani, strengthening digital infrastructure, and implementing structured referral and re-referral pathways [51], that eventually connects the urban tertiary care hospitals only for specific requirements.

## Conclusion

In conclusion, the findings of this study, offers novel insights into physicians’ decision-making processes in urban India. It provides empirical evidence to inform policymakers and healthcare administrators about the systemic gaps and potential areas for intervention. Lastly, the study highlights practical recommendations for developing a structured, patient-centred referral system that can enhance healthcare delivery in urban India. This analysis highlights the informal nature of referral decision-making in a large urban Indian healthcare system and emphasizes the urgent need to address several factors, that necessitates a comprehensive approach. Development of an advanced data management infrastructure connecting patients, primary physicians and specialists, formalization of physicians’ network, training and recruitment of healthcare staff associated with referral mechanism, patient-centric care, the design of standardized communication protocols, and the implementation of robust monitoring and evaluation frameworks can ensure optimization of the referral system that enables patients receive timely and appropriate care, ultimately improving healthcare outcomes and patient satisfaction. The findings of this study will help practitioners, administrators, and policymakers to frame a robust healthcare referral system not only for urban settings, but we believe that if the policy improvements are adapted to rural contexts, it will also benefit rural patients. By developing an efficient and integrated healthcare referral mechanism, India can move towards a more equitable, affordable, and high-quality healthcare system that meets the diverse needs of its population.

This study, while emphasizing patient-centric approach in healthcare referral in India or any other developing country, advances the ‘Social Determinants of Health’ framework to ‘Social Determinants of Healthcare’. This proposition suggests examining the influence of economic status, awareness level, socio-cultural and political ecosystem, and social support of patients on the overall referral dynamics to achieve universal health coverage and Sustainable Development Goal (SDG)-3.

Yet, this study has certain limitations. Referral practices and experiences may differ across specialties. The problems associated with a large capital city in India, may not be generalized for smaller cities and towns. Even though this study is qualitative in nature, the sample selection is limited in terms of lack of representation of rural hospitals and overrepresentation of private healthcare practitioners. Therefore, the study findings cannot be generalized. Additionally, the responses can suffer from social desirability bias during the interviews, which might undermine the presence of the profit angle while suggesting a referral, especially in the private institutions. The policy suggestions will not be uniformly applicable and implementable within short time across geographies considering the vastness of the resource-strapped country. Finally, besides the observed factors there are multiple other factors and their intersectionality that determine referral suggestions and adherence behaviour [52]. Further studies should examine several patient related factors such as negative past experiences, inadequate social support, lack of facilities, and cultural beliefs; while more efforts are required to understand how financial constraints and limited health knowledge among patients can be improved, especially among the underprivileged sections, while facilitating timely care or better adherence with referrals in low- and middle-income countries including India.

## Supporting information

S1 File Qualitative Study Raw Data Set. (XLSX)
